# Surrogate consent to non-beneficial research: erring on the right side when substituted judgments may be inaccurate

**DOI:** 10.1007/s11017-016-9363-y

**Published:** 2016-04-30

**Authors:** Mats Johansson, Linus Broström

**Affiliations:** Department of Medical Ethics, Lund University, BMC I12, 221 84 Lund, Sweden

**Keywords:** Surrogate decision making, Surrogate accuracy, Non-therapeutic research, Non-beneficial research, Substituted judgment, Research ethics

## Abstract

Part of the standard protection of decisionally incapacitated research subjects is a prohibition against enrolling them unless surrogate decision makers authorize it. A common view is that surrogates primarily ought to make their decisions based on what the decisionally incapacitated subject would have wanted regarding research participation. However, empirical studies indicate that surrogate predictions about such preferences are not very accurate. The focus of this article is the significance of surrogate accuracy in the context of research that is not expected to benefit the research subject. We identify three morally relevant asymmetries between being enrolled and not being enrolled in such non-beneficial research, and conclude that when there is a non-negligible probability that surrogates’ predictions are wrong, it will generally be better to err on the side of not authorizing enrollment.

## Introduction

Non-therapeutic research, or more broadly, research that is not expected to provide any benefits to the research subject, raises serious ethical concerns when it involves individuals that cannot provide valid informed consent to their participation. The moral justification for allowing such non-beneficial^1^ research, it is typically acknowledged, must go beyond pointing out that the relevant research is important to those who may benefit from the results. Codes of research ethics incorporate to that effect various safeguards, including requirements of ethical review, limits on acceptable risks and burdens, etc. Part of the standard protection package is also a prohibition against research that has not been authorized by a legal representative, or surrogate decision maker—a requirement most notably expressed in the highly influential *Declaration of Helsinki* (art. 28) [2].

The very point of having a surrogate consider whether or not to consent is to ensure that the prospective research subject’s views and interests are represented when those individuals are not themselves able to express such views or protect those interests. Differently put, surrogates are not supposed to be the vehicles by which research, health care, etc. advance *their* interests. On the contrary, the very function of surrogates is to act as gatekeepers against society not giving due consideration to the interests of the research subject. It is less clear, of course, how a surrogate decision maker best discharges his or her responsibilities towards a decisionally incapacitated individual when it comes to taking a stand on research enrollment. A widely endorsed idea, however, is that surrogates primarily ought to make their decision based on what the decisionally incapacitated subject did want (before the onset of incapacity), or would have wanted had he or she had capacity [3, 4]. From this viewpoint it clearly matters whether surrogates are *accurate* when trying to identify the preferences of individuals lacking capacity, that is, whether the decisionally incapacitated really did or would have the wishes that surrogates believe they did or would have. Empirical studies have addressed this issue of surrogate accuracy with regard to research participation [5–7]. Although there is much to be said about the methodological challenges of empirically assessing surrogate accuracy in general [8–10]—the typical study design is one where potential surrogates are asked to guess what preferences loved ones (with presumed capacity) say they have for various hypothetical scenarios—the studies do indicate that the accuracy is not that impressive, and this has been a source of worry. Accordingly, these results have led some commentators to suggest that we should find ways to improve surrogates’ ability to correctly predict whether individuals lacking capacity would consent to research participation [6, 11].

No doubt, improving upon surrogates’ ability to identify the hypothetical wishes of would-be research subjects is a worthwhile enterprise. However, unless such predictions can be made foolproof (which is unlikely), educational and other efforts to attain greater accuracy would have to be supplemented with principles for determining how surrogates ought to proceed when substituted judgments are uncertain/unreliable. In this article, we explain why the prospects of being right about what the person would want is not the only relevant consideration when a surrogate is to decide whether or not to authorize the enrollment of a decisionally incapacitated research subject under the standard regulatory safeguards. In the context of non-beneficial research, there are morally relevant asymmetries between enrollment and non-enrollment, which also need to be addressed.

## Normatively relevant asymmetries between false positives and false negatives

Imagine a scenario where the surrogate, in the light of the available evidence, is not able to tell at all what the decisionally incapacitated person wanted, or would have wanted if he or she had been able to take a stand on the issue.^2^ Assume that the surrogate therefore estimates that there is a fifty percent chance that this person would have consented to being enrolled in the study. This raises the question whether the surrogate, on the assumption that standard safeguards are in place, could just as well flip a coin? We think not. It not only matters how likely it is that he or she is correct, but also what would happen if the surrogate were to reach a decision based on the wrong prediction.^3^ Two types of mistakes must be considered^4^:

*The false positive*: The surrogate incorrectly predicts that the individual would consent to participate in the research, and bases his or her decision on that prediction.

*The false negative*: The surrogate incorrectly predicts that the individual would not consent to participate in the research, and bases his or her decision on that prediction.^5^

At first glance, these mistakes may appear equally problematic, as both of them consist in reaching a decision that is in conflict with the individual’s preferences. Both mistakes may thus seem to fail to respect the subject’s autonomy^6^. As it turns out, however, the false positive involves additional risks, compared to the false negative. This means that, unless additional relevant asymmetries can be established, the former should in such circumstances appear worse than the latter. Below, three risks will be addressed—relating to harm and burden, instrumentalization, and human rights violations—which together suggest that it may well be better to err on the side of non-enrollment, when as a surrogate one is asked to authorize that a subject is enrolled in non-beneficial research. Those three considerations are broadly agreed to be morally relevant when it comes to human subjects in research in general, and unless there are specific reasons to the contrary, the assumption has to be that they ought to be important also in surrogates’ protection of the decisionally incapacitated. The asymmetries that we have in mind should be most easily grasped in the case of total uncertainty, which is why this will initially be the assumed scenario. We maintain, however, that the basic thrust of the argument remains valid also in cases where the likelihood of error is smaller, even though the practical impact of the argument may need to be qualified in some of those cases. We shall return to the relevant qualifications in the last section.

### Harm and burden

When there are no expressions of will in advance, and there is no evidence allowing the surrogate to reconstruct what the individual would have wanted, the main option that remains is to consider what would be in the “best interest” of the person concerned. Just what is in the best interest of a person is obviously a complex and controversial issue. In research, for example, one may face the question of whether the research subject will benefit clinically from being part of a study, whether the fact that the research subject will receive extended health monitoring may outweigh expected burdens of participation, or whether weight should be given to the possibility that the subject will subsequently feel good about having been part of progress being made in the relevant field. As indicated, we believe there is no good reason to rule out of consideration benefits for the research subject that are not strictly clinical; for present purposes, we only aim to exclude from the class of theoretically possible benefits the satisfaction of the individual’s preferences, or respect for his or her will, as its inclusion would partly collapse the distinction between substituted judgment and best interests.

The present argument, however, does not depend on specific views about what kinds of benefit might be worth exploring in research contexts. Whether there is a significant likelihood of some clinical, psychological, or other kind of benefit should be open for debate on a case-by-case basis. While we do believe that the burden of proof in any given case is with those who claim that there are significant enough prospects of participant benefits, our present argument is based only on the observation that research often does not offer any benefit to research subjects and that we need to sort out what principles ought to govern surrogate decisions about research participation in those cases. We thus make no claims concerning research participation in general, but only make claims about the asymmetries related to research acknowledged to be non-beneficial.^7^

Not expecting that a research subject will benefit from participating in a certain study only provides half of the picture, when deciding on whether one mistake about preferences is worse than the other on grounds of wellbeing. Potential downsides to being enrolled must also be considered. Medical research typically introduces some risk of harm to the participants, and virtually all human trials involve some burden or discomfort, albeit very slight.^8^ If nothing else, participating typically takes time. Examinations, surveys, and follow-ups may take only a couple of minutes, but they may, depending on the nature of the research, take several hours, and research involvement frequently entails a number of intervention sessions. In addition, medical research often deals with sensitive personal data, in ways that may threaten the privacy of the individuals concerned.

Hence, it will generally *not* be in the best interest of any particular individual to be enrolled in non-beneficial research. The point is not that typical burdens are especially damaging; they need not be at all. It does not matter, however, that the risks and burdens are expected to be minimal, or close to that.^9^ On anyone’s account, minimal risks and burdens include risks of harms and burdens that one would have wished that someone one cares for would not have to endure, unless he or she had something to gain. If there is no expected benefit, it will surely be in the interest of a person not having to endure even slight and temporary pain, such as having one’s blood taken. In addition, there is still no consensus on just what the notion of minimal risk and burden is supposed to cover [15–17]; hence a surrogate who takes his or her protective role seriously would do well, for reasons of precaution, to refuse enrollment whenever there is uncertainty as to the true meaning of “minimal risk and burden” in a given (non-beneficial) study. Furthermore, analyses of research risks are typically based on the harms and burdens expected if everything goes to plan, but the possibility of human oversight in the practical conduct of research remains, adding another layer of uncertainty to be considered by the surrogate. If there is significant uncertainty as to what the individual lacking capacity would have wanted, a surrogate decision maker would thus seem to be obligated to refuse authorizing research enrollment in what has been agreed to be non-beneficial research. This would simply be the safer bet, or so it seems, with respect to the subject’s best interests.

### Instrumentalization

The main purpose of conducting research is to promote certain interests, but those do not (other than by accident or secondarily) coincide with the interests of the research subject. Research aims at generating knowledge of more general value, which may or may not be of any use in benefiting the research subject. This difference between (non-beneficial) research and activities tailored to profit those directly involved implies that non-beneficial research risks *instrumentalizing* decisionally incapacitated research subjects, i.e., using them merely for the benefit of others. As with regard to perhaps most key concepts in moral philosophy, there is no agreement on what exactly instrumentalizing someone, using someone merely for the benefit of others, amounts to, or should amount to. But there are two distinct basic lines of thought about how instrumentalization can be avoided.

First, when an individual is sufficiently informed about what is at stake, and autonomously, without being coerced or acting from a submissive position, consents to participation in non-beneficial research, this individual can be said to use him- or herself as a means to others’ ends, and should not be considered instrumentalized in any morally interesting sense. While consent, or self-determination more generally, may be associated with other values or purposes as well, instrumentalization is one threat it is typically acknowledged to protect against. For the decisionally incapacitated, such consent is, by definition, not possible, and thus, they do not enjoy this particular protection against instrumentalization. But certain deviations from the relevant autonomy ideal—lesser substitutes along the autonomy dimension, as it were—may still imply that sufficient consideration has been given to the preferences or perspective of the individual concerned, for him or her not to be instrumentalized. Notably, it has for example been argued that we can trust that people are not used merely as means in the morally relevant sense to the extent that we can trust that we proceed in accordance with accurate substituted judgments [18].

Second, with respect to a person incapable of making autonomous enough choices, and regardless of whether he or she would have consented, when we see to it that “our treatment of this person is governed or guided in sufficiently important ways by some relevant moral belief or concern [for the well-being or moral claims of the person so treated],” neither then do we seem to use this person merely as a means to others ends [19]. Clarifying what the “sufficiently important ways” are is precisely the sort of thing moral philosophers will struggle with. But, for example, when a toddler is fed at certain times because this is believed to be good for her, this child is presumably not instrumentalized, even though the decision is made *for* her, not by her, and regardless of whether it would make sense to claim that there is hypothetical consent to that act. However, when there is no consent or any legitimate substitute for consent, securing respect for individual autonomy, and when the intervention is before all else guided by *scientific* concerns, there is a prima facie case for saying that such a research subject is instrumentalized.

It is one thing to say that involving the decisionally incapacitated in non-beneficial research may instrumentalize those individuals; it is another thing to say that instrumentalization should be a *concern*. That there is something morally wrong with instrumentalization is an idea that philosophically goes back primarily to Immanuel Kant, but it seems deeply entrenched in ordinary moral thinking as well. The main worry here is not that research participation will tangibly disadvantage in some way the person enrolled, even though the risk of being harmed or burdened is likely to be greater (all else being equal) in any endeavor that is not primarily meant to benefit or protect the person involved. The main concern is that the individual will be otherwise *wronged*, by being treated in a way that conflicts with his or her dignity, or in a way that conflicts with what any person, by virtue of his or her personhood, deserves. No doubt, these ideas involve concepts and assumptions that are in need of clarification and justification, respectively. The notions of dignity and personhood are notoriously elusive, and whether all individuals lacking capacity should be considered persons in the relevant sense, or as having dignity that needs protection, has occasionally been questioned.^10^ Nonetheless, it has been a long-standing and widespread worry that when researchers make use of those who cannot validly consent to their research participation in studies agreed not to promise those individuals any benefits, those researchers exhibit a lack of respect that is morally objectionable, regardless of whether the research participants will suffer in some other way.

One might of course object that if the researcher’s intention is noble and attitude not exploitative—if, for instance, he or she honestly seeks to enroll only people who would have approved, but mistakenly also enrolls an individual who would not have done so—the research subject is not instrumentalized. In that case, it might seem, the researcher just happens, as a matter of bad luck, to act in conflict with what the research subject in question would have wished. Certainly our notion of instrumentalization, or at least one common form of the notion, is essentially about the *attitude* with which certain choices are made, more so than the actual effects. But even on that understanding, there is a risk of instrumentalization to consider, because the more one acknowledges the risk of being wrong, when one authorizes enrollment of the decisionally incapacitated individual in non-beneficial research, the more one seems *indifferent* to whether or not this person will be used merely as a means to others’ ends.

Anyway, for present purposes, we need not get to the bottom of in what exactly instrumentalization consists, exactly why it would be morally questionable, or exactly how questionable it would be. It is sufficient to note that there is a long-standing worry among ethicists that people are instrumentalized when they are subjected to certain kinds of research interventions, and that, for all that we know, there might be something to that worry, even if we have not yet elaborated it in fully satisfactory ways. Because, regardless of how one might wish to elaborate this familiar notion of instrumentalization, it is obvious that the choice *not* to enroll a decisionally incapacitated individual in a research study is not a choice that instrumentalizes this person, all else being equal—clearly he or she is not at all used by the researcher, for any purpose. The point, in other words, is not that there is a crystal clear notion of illegitimate instrumentalization, such that it has been conclusively shown that decisionally incapacitated individuals are instrumentalized in that sense when they are enrolled in non-beneficial research. Rather, the point is that there is a non-negligible risk of such instrumentalization in this context, whereas non-enrollment in this respect will be the safe choice, as this option *obviously* is not associated with a risk of instrumentalization.

### Rights

A third difference between the two mistakes (the false positive and the false negative) is that only one of them seems to lead to an outcome that may violate acknowledged human rights. Enrolling someone in research, especially studies in the field of medicine, will, as a rule, infringe upon that person’s bodily or psychological integrity in ways that would normally be regarded unacceptable unless the individual provided valid informed consent. Recognition of this negative right is uncontroversial in the human rights tradition, as articulated in, e.g., article 7 of the *International Covenant on Civil and Political Rights* [21]. “In particular,” the article says, “no one shall be subjected without his free consent to medical or scientific experimentation.” Consent, accordingly, plays a central role in international conventions as well as national legislation informed by human rights. When individuals lack decision-making capacity, valid consent is not possible, but the search for a good enough substitute in the form of accurate conjectures about their (hypothetical) preferences is an implicit acknowledgment of the right under threat, or so it would seem.

The point here, it should be stressed, is not that the relevant right ought to be regarded as absolute. Like with any other recognized right, it may well have to give way to the satisfaction of other, ultimately stronger, rights of the individual concerned or of others. For example, other people’s right to health might require research that necessitates the violation of a research subject’s right not to be experimented on without consent. The point is just that there is an acknowledged right under threat, whether that right is defeasible or not. And the existence of such a right does not depend, we should add, on the right holder having decision-making capacity. It may seem pointless to uphold certain other rights, such as the positive right to education, if individuals, due to lack of capacity, have no chance whatsoever to reap the fruits of exercising those rights. The moral right not to be subjected to experimentation without consent is not like that, however, since even in cases where the required capacities for valid consent are absent, there is an obvious purpose to protecting the individual’s bodily or psychological integrity.

Is there, then, a corresponding right to be enrolled in research? For what it is worth, no such right is mentioned in contemporary codes and conventions. And neither would it make much sense to recognize a general claim to being offered to participate in research. For one thing, it is hard to see what strong enough individual interest would be served by the recognition of such a right. Second, in order for an individual to have a legitimate claim to being enrolled in research, there would have to be an obligation on the part of the researcher to enroll that individual. But surely, even if the case could be made that a researcher has an obligation to conduct a particular study, he or she would have no obligation to enroll every individual in that study. Were research to be organized so as to respect everyone’s alleged right to participate in it, it would bear little resemblance to the enterprise we know today, since the very point of research is to serve other purposes, recognized to be valuable by society. After all, subjects are invited to participate in research not because they want to participate, or because they will benefit from doing so, but because researchers need data. The requirement of consent is in this context a protective measure, not a practice aimed at promoting individual autonomy.

Obviously there may be a right to be included in research that offers a potential benefit to the subject, for example, by means of experimental treatment, as David Wendler has suggested [18]. This might simply be subsumed under the right to health. Such a right would however be irrelevant to the issue at hand, as we are here only concerned with non-beneficial research. Another idea, also mentioned by Wendler, is that a right might be violated when subjects are excluded from research without good reason [18]. If a research subject is the victim of discrimination, for example, it constitutes, by definition, a violation of the right to equal treatment. Discrimination, however, is differential treatment that is not based on any morally relevant differences between the individuals in question, and the choice to exclude precisely those who cannot protect their own interests due to decisional incapacity arguably does not fit that bill. *The United Nations Convention on the Rights of People with Disabilities*, of course, with its article 12 requiring equal recognition before the law, will (if interpreted along the lines of General comment 1) find it discriminatory not to allow everyone, regardless of cognitive ability, to make up their own minds regarding research participation [22]. That, however, would be a different thing entirely, as from this perspective, the premise of the whole discussion—that sometimes surrogate decision makers may have to make decisions about research enrollment of decisionally incapacitated individuals—is rejected. All in all, there simply is no acknowledged right to be enrolled in research that will be violated when individuals admitted to be decisionally incapacitated are excluded, whereas there is a generally affirmed right not to be experimented on without consent—a right clearly at stake when judgments about the individual’s wishes are uncertain or unreliable.

## Implications

In a framework where respecting the decisionally incapacitated individuals’ hypothetical wishes regarding research participation takes central place, but where there is also a non-negligible probability that surrogates may be wrong about those wishes, we need principles that help us determine what mistakes would be the worst to make. We have made the case that with respect to non-beneficial research, there are important asymmetries between the two mistakes that the surrogate can make: false positives and false negatives. These asymmetries, in turn, would seem to support a particular piece of *precautionary* reasoning. Surrogates, unless special circumstances suggest otherwise, should refuse to authorize research enrollment in those situations in which there is a significant risk that a mistake about the individual’s preferences will be made.^11^

What would the implications of this proposal be from a societal viewpoint? Assume that based on precautionary reasoning, surrogates would systematically refuse to authorize enrollment in non-beneficial research. Under current provisions, this would imply that many seemingly valuable studies could not take off. This, in turn, would in all likelihood imply that there will be little scientific progress on many medical and other problems plaguing large and vulnerable patient populations. There are good grounds for taking measures to ensure that this scenario will not materialize. It would be a scenario in which many people are significantly deprived of wellbeing that they could have been offered, and one in which certain groups of future patients could possibly be said to be victims of discrimination. There are thus both consequentialist and egalitarian reasons for allowing at least some non-beneficial research on research subjects lacking capacity.^12^ However, the present discussion does not concern whether society should have a policy where such research is made possible; it concerns what moral grounds there are for surrogate decision makers to authorize research enrollment in any individual case. Society could certainly choose to restrict surrogates’ mandate under the relevant circumstances, for example, by making room for exceptions to the requirement of surrogate consent. In this article, we express no views on this option or, for that matter, on how society in general should approach the problem.

Our point is just that when surrogate predictions about preferences regarding research participation may prove inaccurate, one needs to assess the relative seriousness of false positives and false negatives, and that surrogates who exercise caution may well have to err on the side of non-enrollment. As already stated, in suggesting this, we obviously assume that society does not call upon surrogates to protect other parties’ interests too, in addition to those of the decisionally incapacitated would-be research subject. It is central to the notion of a surrogate that he or she is given the responsibility to serve the interests of the incapacitated individual, and not anyone else’s. Were surrogates to be asked to balance the interests of this individual against the interests of a greater population, of society or some other party, they would rather turn into a one-person ethical review board. That would make their role utterly unclear and, moreover, point to the need of a *true* surrogate—someone whose job it is to protect the decisionally incapacitated individual’s interests in particular.^13^

Have we considered that many people may be inclined to give their prospective surrogates a very broad mandate? There is actually empirical support not only for the assumption that many people seem to be prepared to give surrogates leeway in making decisions about research participation on their behalf, but also for the fact that this willingness to let surrogates deviate from what they would state as their own preference may actually be strengthened after “democratic deliberation” [12]. While interesting, these findings have no bearing on the present point. In fact, it would not even matter if one were to find out that surrogates would generally be prepared to allow leeway. First, uncertainty will remain as to whether, in the case at hand, the decisionally incapacitated individual would have given his or her surrogate (some or complete) leeway; the possibility of erring on a wrong side remains. Second, even if it were known with certainty that the individual concerned did or would allow the surrogate leeway, this information alone would offer no guidance as to what the surrogate ought to decide. The surrogate would know that the individual was or would be okay with any responsibly made decision on the issue of research inclusion, but he or she still would not know what that decision should be.

Now, just what precaution dictates obviously depends on the relative weight given to the various values involved, and on the probabilities of them being realized. For expository reasons, we have mainly looked at the scenario where surrogate accuracy may not be greater than chance, but in many cases, surrogates could obviously do better than that. Imagine for purposes of argument that numbers do make sense: the chance of being right about what the incapacitated individual would have wanted could be, say, 60/40, or even better, 90/10, or any other estimate. For someone who assigns a significantly greater value to satisfying a decisionally incapacitated person’s hypothetical preferences than to safeguarding rights, non-instrumentalization, and non-exposure to risks and burdens, a significant chance of being right that this person would have wanted to take part in the study may well be considered sufficient in any or all of those circumstances for authorizing enrollment; the argument of this article certainly leaves this open.

To repeat, however, for any conclusions to be drawn about the legitimacy of allowing the incapacitated individual to be part of a study, accuracy assessments would have to be supplemented with evaluations of the seriousness of being wrong in various ways. Unless surrogates could be considered completely certain about what the individual would have wanted, that is, one would need some kind of precautionary principle to provide the guidance that the basic decision-making standards do not offer on their own. And more specifically, the greater the uncertainty, the greater the relative value of the relevant kinds of autonomy would need to be, given that false positives are associated with various downsides. Again, we have not, in this article, attempted to establish what reliability level would be enough to warrant authorizing inclusion. But anyone who believes that the value of satisfying the individual’s prior or hypothetical wishes regarding research enrollment is so great that surrogates ought to err on the side of inclusion in any case in which it is more likely than not that he or she would have wanted to be enrolled certainly has a burden of argument. Such a person quite likely faces an uphill struggle, in our view, given not only that the moral force of prior or merely hypothetical wishes is contested but also that the positive choice of participating in research is something to which we are not generally afforded an autonomy right.

## Footnotes

1 We adopt the term from David Wendler [1]. Classifying research as either beneficial or non-beneficial obviously does not reflect the fact that studies may often contain both beneficial and non-beneficial elements. Greater precision in this regard should not be needed, however, in order to understand and evaluate the argument of this article.

2 For the purposes of the following discussion, there will be no need to distinguish between the distinct aims of respecting the individual’s past wishes (when he or she had decision-making capacity) and respecting his or her hypothetical wishes, respectively, and for the most part, we will frame the discussion in terms of the latter (substituted judgment).

3 Acting in conflict with a person’s wishes does not amount to the same thing as making a decision that is in conflict with that person’s wishes. In what follows, however, the decision will be assumed to result in an action congruent with the prediction of the subject’s preferences.

4 It should be noted that surrogate predictions could concern another kind of preference, namely, whether the subject has (or would have) given the surrogate some leeway when deciding whether or not to approve enrollment—a preference that many people actually seem to share [12–14], and one that is addressed later in this article.

5 Notice that *not consenting* includes the possibility that the person has no opinion whatsoever. Not saying yes is, after all, not the same as saying no.

6 Julia T. Newman and colleagues take only false positives to threaten the ethical principle of autonomy for the patient [5]. We take this to be misleading since *both* scenarios fail to satisfy the principle of autonomy.

7 In many real-world situations, there may indeed be some uncertainty as to whether the research subject will benefit from being enrolled. While the study perhaps is not designed to be therapeutic, for example, it nonetheless may bring with it some collateral advantages to the participant, if conditions are fortunate enough. To what extent a possible, but more unlikely than likely, benefit should be taken into consideration in enrollment decisions under uncertainty is a good question, but it falls outside of the scope of this article. That future discussion, however, will in a way merely be an extension of the points made in the present one, about the need to evaluate the seriousness of the different mistakes that can be made.

8 Obviously there are some exceptions, one being cases where decisional incapacity is due to lack of consciousness.

9 Contemporary codes of research ethics typically state that non-beneficial research on subjects lacking capacity may not be conducted if those subjects are exposed to more than minimal risks or burdens. See *The**Declaration of Helsinki*, art. 28 [2].

10 For example, on Kant’s own theory, only rational individuals can be instrumentalized in the morally troublesome way mentioned above. Although some commentators accept this consequence [20], and exclude individuals without decision making capacity from their analysis, we see no reason to be faithful to Kant’s own ideas in the present context. Instead, we find it more reasonable (although not self-evident) to regard any sentient being as at risk of being instrumentalized.

11 There are indications that surrogates’ predictions of preferences regarding participating in research that involve risks greater than minimal are not much more reliable than a coin flip [11].

12 Some commentators have argued that it is only fair, under certain circumstances, that subjects lacking capacity participate in research, given what they receive from the society [23]. A more radical thesis is defended by John Harris and Søren Holm [24], according to which, roughly, surrogates and society would protect the interests of the decisionally incapacitated by discharging the latter’s (alleged) moral obligations to participate in research. This proposal is obviously problematic for a number of reasons, and would have far-reaching implications.

13 As suggested by an anonymous reviewer, it is an interesting empirical question to what extent surrogates feel that they ought to take researchers’ or society’s interests into consideration, and if so, whether this belief may actually affect the accuracy of their substituted judgments.
